# Response to patient safety incidents in healthcare settings in Ghana: the role of teamwork, communication openness, and handoffs

**DOI:** 10.1186/s12913-023-10000-0

**Published:** 2023-10-06

**Authors:** Collins Atta Poku, Priscilla Yeye Adumoah Attafuah, Emmanuel Anongeba Anaba, Patience Aseweh Abor, Edward Nketiah-Amponsah, Aaron Asibi Abuosi

**Affiliations:** 1 Department of Nursing, College of Health Sciences, Kwame Nkrumah University of Science and Technology, Kumasi, Ghana; 2 Public Health Nursing Department, School of Nursing and Midwifery, University of Ghana, Accra, Ghana; 3 Department of Population, Family and Reproductive Health, School of Public Health, University of Ghana, Accra, Ghana; 4 Department of Public Administration and Health Services Management, University of Ghana Business School, Legon, Accra Ghana; 5 Department of Economics, University of Ghana, Accra, Ghana

**Keywords:** Communication openness, Handoffs, Patient safety incidents, Response, Teamwork

## Abstract

**Background:**

Patient safety incidents (PSIs) in healthcare settings are a critical concern globally, and Ghana is no exception. Addressing PSIs to improve health outcomes requires various initiatives to be implemented including improving patient safety culture, teamwork and communication between healthcare providers during handoffs. It is essential to acknowledge the significance of teamwork, communication openness, and effective handoffs in preventing and managing such incidents. These factors play a pivotal role in ensuring the well-being of patients and the overall quality of healthcare services.

**Aim:**

This study assessed the occurrence and types of PSIs in health facilities in Ghana. It also examined the role of teamwork, handoffs and information exchange, and communication openness in response to PSIs by health professionals.

**Methods:**

A cross-sectional study was conducted among 1651 health workers in three regions of Ghana. Using a multi-staged sampling technique, the Survey on Patient Safety Culture Hospital Survey questionnaire and the nurse-reported scale were used to collect the data and it was analysed by descriptive statistics, Pearson correlation, and linear multiple regression model at a significance of 0.05.

**Results:**

There was a reported prevalence of PSIs including medication errors (30.4%), wound infections (23.3%), infusion reactions (24.7%), pressure sores (21.3%), and falls (18.7%) at least once a month. There was a satisfactory mean score for responses to adverse events (3.40), teamwork (4.18), handoffs and information exchange (3.88), and communication openness (3.84) among healthcare professionals. Teamwork, handoffs and information exchange and communication openness were significant predictors of response to PSIs, accounting for 28.3% of the variance.

**Conclusions:**

Effective teamwork, handoffs and information exchange, and communication openness in the healthcare environment are critical strategies to enhance PSI response. Creating a culture that encourages error response through teamwork, communication and handoffs provides healthcare professionals with opportunities for learning and improving patient outcomes. Training programs should therefore target health professionals to improve patient safety and competency. Through the implementation of evidence-based practices and learning from past incidents, the healthcare system will be able to deliver safe and high-quality care to patients nationwide. Patient safety must be recognized as an ongoing process. Therefore, a meaningful improvement in patient outcomes requires all stakeholders’ commitment.

## Introduction

Patient safety incidents (PSIs) are unintended and harmful consequences of medical treatment or care. In high-income countries (HICs), an estimated 10% of patients experience PSIs while in low- and middle-income countries (LMICs), the rates may be even higher due to a lack of adequate resources and systems for patient safety [1]. It ranges from minor side effects to serious injuries and even death. It is regarded as a major concern because it may adversely impact hospitals’ patient safety and the quality of healthcare delivery [2, 3].

Incidents from iatrogenicity rank among the three leading causes of death in HICs [4]. It can also have serious consequences for patients, including prolonged hospital stays and complications including disabilities. The PSI incidence is alarming in that up to 1.1% of hospital admissions result in death [2, 5]. Moreover, the high financial costs associated with PSIs burden the healthcare system and can lead to increased healthcare costs for everyone. According to Mikos et al. [6], the estimated annual cost of PSIs is 17.1 billion dollars. The two most frequent types of PSIs (pressure sores and post-operative infections) alone account for the largest portion of the costs at 6.5 billion dollars. Catheter infections, infections resulting from transfusion and infusion sites, injections, and similar procedures also result in significant extra healthcare costs, totalling over one billion dollars [7, 8].

Healthcare providers must be conscious of PSI risks and take steps to prevent them as much as possible. Most healthcare organizations have, therefore, initiated and prioritised patient safety strategies to prevent PSIs from occurring. This includes implementing evidence-based practices, improving communication among healthcare team members, and investing in quality improvement efforts [9]. Providing staff training, and continuously monitoring and reviewing care processes have also been highlighted as ways to improve patient safety efforts [10, 11].

Besides these strategies, health systems play a crucial role in reducing PSIs in patients. This is done by investing in resources and implementing systems to quickly respond to patient harm. Measuring and reporting PSIs raises awareness of potential errors and promotes a safety culture. An effective response to PSIs not only remedies problems but also provides a surveillance process that helps identify risks and improve patient and staff safety [12, 13]. One way to do this is by implementing a quick response system to PSIs and conducting retrospective analyses to understand the root causes of these events. This can help healthcare providers identify patterns and trends and mitigate risks of similar events in the future [14, 15].

Additionally, measuring and reporting PSIs can help raise awareness of potential errors and promote a safety culture within the healthcare system. By paying attention to PSIs, healthcare systems can address problems as they arise. Moreover, healthcare organizations can also identify and assess patient and staff risks of PSIs and minimize them [16].

It has been established that having an effective response system in place for reporting and addressing vulnerabilities in healthcare systems can promote resilience and prevent further harm [17]. Nevertheless, such a response system can harm the positive campaign on PSIs by reducing open reporting and discussion of mistakes. This can hinder efforts to improve care quality. In effect, negative responses from managers to PSIs occurrence can also create a culture of fear around reporting and discussing mistakes. This can hinder patient safety efforts [18, 19]. It is important to encourage open and honest discussion of PSIs to continuously improve patient care. This can be achieved through effective healthcare systems to respond to vulnerabilities and incidents through incident reporting policies and tools. This will help identify and address problems as they arise [20]. These proactive efforts when implemented can also optimize care delivery to promote resilient healthcare systems [21].

Teamwork has been identified as indispensable for safeguarding patient safety and promoting healthcare quality [12]. This can involve implementing strategies such as regular team meetings, effective communication practices, and shared decision-making processes to promote collaboration and coordination among healthcare staff [22, 23].

It is also vital to ensure healthcare team members feel comfortable speaking up and voicing concerns. This can help identify and address potential problems before they lead to adverse events. Overall, promoting a culture of teamwork and continuous improvement enhances patient safety practices, especially reporting incidents [24–26].

Teamwork among healthcare staff is critical to improving PSI response. Teamwork perceptions of healthcare professionals play a significant role in improving adverse events reporting rates [27], patient outcomes [28], enhancing job performance in healthcare teams [29–31], and overall patient safety in healthcare facilities [31, 32]. A human factors approach, which considers healthcare professionals’ physical, cognitive, and social characteristics, helps to identify and address potential obstacles to teamwork. This ensures that all team members can effectively contribute to patient care [33].

Additionally, in a system as multifaceted as healthcare, collaboration within and across organizations through teamwork and communication has reduced the amount of the health workforce’s contribution to PSIs in about 20% of cases [34, 35]. This is achieved by ensuring that all parties involved in a patient’s care know the patient’s medical history and treatment plan. This can reduce the risk of misdiagnosis, medication errors, and others [36, 37].

Handoffs make information and responsibilities between healthcare practitioners possible, which are a crucial part of the healthcare industry [38]. Because inadequate communication leads to multiple difficulties, it has long been recognized that the transfer of patient knowledge, professional responsibility, and accountability between caregivers presents a potentially difficult period for patient safety [39]. The second Institute of Medicine (IOM) report, *Crossing the Quality Chasm*, highlighted the need for standardization and accountability in handoffs to ensure that the transfer of care is smooth and that patient safety is not compromised [40]. Standardization can be achieved through structured handoff protocols and tools, such as checklists and electronic medical records. These protocols help to ensure that all necessary information is shared and responsibilities. When combined with accountability, standardization helps minimize communication errors during transfers of care. This has been shown to promote patient safety through positive responses to PSIs [41].

Hospitals and other healthcare organizations need open communication and a safety culture to promote PSI reporting. When staff feel able to speak openly about safety concerns and PSIs, it can help create an environment where issues can be addressed promptly. This can lead to improved patient safety [42]. This can be achieved through a variety of strategies, including promoting open communication at the unit level. In addition, it provides opportunities for staff to report concerns and creates a culture of transparency and accountability. By fostering an open communication and safety culture, healthcare organizations can prevent PSIs and improve patient outcomes [43].

Until this study, PSIs have not been studied extensively in healthcare settings, as well as teamwork, effective handoffs, and communication openness among health professionals in Ghana. The study’s findings will help identify innovative protocols and best practices to minimize adverse events. Moreover, it will provide healthcare organizations with strategies for optimizing teamwork and communication. Healthcare networks can share these findings to improve patient safety practices continuously. The study, therefore, assessed PSI occurrence in healthcare facilities. It examined the role teamwork, handoffs and communication openness play in healthcare professionals’ responses to PSIs. The findings from the study may be used to improve the safety culture in Ghana’s healthcare system by reducing PSIs to improve quality care.

## Methods

### Study design and setting

A cross-sectional survey using health professionals in 13 healthcare facilities in Ghana was employed. This approach provided a snapshot of health professional views on the phenomena under study at the period of the study. It also offered a valuable understanding of the current state or prevalence of PSIs in hospitals and the factors that affect them, which provided valuable insights into relationships and disparities among the health workforce [44]. The study was conducted in three regions in Ghana (Bono, Greater Accra, and Upper East) selected randomly from the southern, middle, and northern ecological zones. The total number of healthcare facilities in Ghana is 1044 and the total health workforce is 122,182 [45]. The healthcare facilities in Ghana include hospitals, health centres, clinics, and community-based health planning and services (CHPs) compounds [45, 46]. Of the selected regions, the Greater Accra region has the highest number of health facilities (438) with the corresponding highest number of health professionals, followed by the Upper East region (211) and the Bono region (120). A total of 13 healthcare facilities were selected for the study, with four facilities each chosen from the Bono and Upper East regions and five facilities chosen from the Greater Accra region. The selection of these facilities was based on the diversity of the working environment across different levels of care. A teaching hospital was also included in the study due to the availability of specialized services.

### Study population

The population included a variety of healthcare professionals from different disciplines, including nurses, doctors, pharmacists, laboratory technicians, and administrative staff from the study sites. To be eligible for inclusion, participants had to be full-time health workers with more than a year of working experience, and they had to agree to participate. Healthcare workers who were on leave were excluded from the study.

### Sample and sampling technique

A sample size of 1651 healthcare professionals was estimated using the Cochran formula [47]. This study used a multi-stage sampling approach, which involves multiple levels of sampling to select a sample from the population. In the first stage, a simple random sampling was used to select three (3) regions from the 16 regions in Ghana. Four (4) hospitals were randomly selected from each of the chosen regions, meanwhile, a Teaching hospital in Greater Accra was added to the selected hospitals, totalling 13 hospitals. In the second stage, a proportionate stratified sampling was used to allocate the sample for each of the 13 hospitals. The distribution of the workforce was as follows Greater Accra region (73,309), Upper East region (26,880) and Bono region (21,993). In the third stage, the convenience sampling method was used to select the participants from the study sites. This approach was considered appropriate for the study.

### Measures

#### Socio-demographic characteristics

This study collected information about the participants’ socio-demographics: age, gender, education, marital status, field of work, job title, working hours, and work experience.

#### Patient safety incidents

The frequency and occurrence of PSIs were assessed using the Adverse Patient Events Scale (APES) [48]. The Scale had the following types of PSIs: medication errors, pressure ulcers, patient falls, physical restraint for more than 8 h, wound infections, infusions and transfusion reactions and complaints from patients and/or family. Participants rated each item according to the frequency with which they occurred during their shifts using a five-point Likert scale (0 = never to 4 = several times a year). Previous studies have revealed that this scale’s internal consistency has Cronbach alpha scores between 0.81 and 0.93 [49]. The current study’s Cronbach alpha value was 0.91.

#### Patient safety culture dimensions

The Survey on Patient Safety (SOPS) Culture, Hospital Survey questionnaire (version 2.0) was adapted from the Agency for Health Research and Quality for data collection [50]. Three (3)  dimensions were adapted from the questionnaire to measure teamwork, communication openness and handoffs and information exchange among healthcare professionals [50]. The new scale included: teamwork (3 items); communication openness (4 items) and handoffs and information exchange (3 items). All the items were on a Likert scale ranging from 1 (strongly disagree) to 5 (strongly agree). The composite mean score of each dimension was computed with a score of ≥ 2.5 indicates an adequate response to the dimensions. The scale has an acceptable reliability score of at least 0.84 and has also reported good discriminant and convergent validity in other studies [51, 52]. The Cronbach alpha value for the scale in this present study was 0.81. Data from hospital units were combined and examined by Agency for Healthcare Research and Quality (AHRQ) survey methods.

### Data collection

This study collected data from the participants over three months, from July to September 2021. To ensure a maximum response rate, the researchers worked closely with the hospital administration to plan and coordinate data collection. We developed a comprehensive survey designed to meet the study’s purpose. Participants were informed about the purpose and significance of the study and written consent was obtained before administering the questionnaire to them during morning and afternoon shifts. The participants were asked to fill out the questionnaire, either at home or at work at their convenience. The distributed surveys were tracked and collected after participants had filled them. The daily received completed questionnaire was kept safely by researchers to ensure confidentiality.

### Data analysis

The SPSS (Version 26.0) was used for data analysis and descriptive statistics were applied to examine the socio-demographic characteristics of the data, types and occurrence of PSIs, and response to PSIs using frequencies, mean and standard deviations. A linear regression analysis model was used to determine the predictive effects of teamwork, handoffs and information exchange and communication openness on response to PSIs after a Pearson Moment Product Correlation analysis was conducted between the predictors and the dependent variable. The test ensured that the assumption of homogeneity of variance and multicollinearity was not violated. The analysis was conducted at a p-value of 0.05.

## Results

### Socio-demographic and work characteristics of participants

Of the 1701 health professionals who received the survey, 1651 (86.2%) responded as summarised in Table 1. An average age of 33.60 years (SD: 6.38) was recorded with more than half being females (55.2%, n = 912). More than 40% worked in medical-surgical units while more than half (54.9%, n = 907) have worked between 2 and 6 years. The majority of the participants (72.5%, n = 1197) were nursing staff. Approximately half of the participants work 30 to 40 h per week.

**Table 1:** Socio-demographic and work characteristics of participants

Socio-demographic data	n	%	Mean	SD
Age			33.60	6.38
Female sex	912	55.2		
Primary Unit/Department				
Medical-Surgical	678	41.1		
Obstetrics and Gynaecology	292	17.7		
Emergency and ICU	108	6.5		
Paediatric/Child Health	141	8.5		
Psychiatry/Behavioural Health	184	11.1		
Diagnostics and Pharmacy	128	7.8		
Administration and Support Staff	120	7.3		
Duration at the unit				
Less than 2 years	452	27.4		
2–6 years	907	54.9		
More than 6 years	292	17.7		
Profession group				
Nursing staff	1197	72.5		
Medical officers	175	10.6		
Others Clinicians (pharmacist, lab etc)	141	8.5		
Managerial, Admin. and Support Staff	138	8.4		
Hours of hours per week				
30 to 40 hours per week	910	55.1		
More than 40 hours per week	741	44.9		

### Perceived occurrence of Patient Safety incidents

As reported in Table 2, there was a prevalence of PSIs in Ghanaian hospitals, as close to a third of the participants (30.4%, n = 502) reported experiencing medication errors at least once a month. Though more than a third (33.7%, n = 556) of the participants have not had their patients experiencing pressure ulcers at their unit, an estimated 21.3% (n = 351) reported experiencing patients with pressure sores once a month. About a third (n = 523, 31.8%) have experienced patient fall in their unit at least several times in a year. Though the use of restraints in healthcare facilities is not a common occurrence in Ghana, nonetheless, approximately half (n = 823, 49.8%) of the participants reported never experiencing it in their units. About 384 (23.3%) and 407 (24.7%) of the participants reported wound infections and infusion /transfusion reactions respectively in their units at least once every month. Patient and/or relative complaints were the commonest PSIs as 33% (n = 545) of the participants reported it occurrence every day.

**Table 2:** Perceived occurrence of PSIs in the unit or work area

Types of PSIs	Occurrence	n	%
Medication error	Never happened Everyday Several times a week Once a month Several times a year Don’t know	348 102 118 502 238 343	21.1 6.2 7.1 30.4 14.4 20.8
Pressure ulcer	Never happened Everyday Several times a week Once a month Several times a year Don’t know	556 35 87 351 162 460	33.7 2.1 5.3 21.3 9.8 27.8
Patient falls	Never happened Everyday Several times a week Once a month Several times a year Don’t know	642 97 54 308 161 390	38.8 5.8 3.3 18.7 9.8 23.6
Physical restraints for more than 8 h	Never happened Everyday Several times a week Once a month Several times a year Don’t know	823 46 51 191 60 480	49.8 2.8 3.1 11.6 3.6 29.1
Wound infections	Never happened Everyday Several times a week Once a month Several times a year Don’t know	370 102 67 384 255 473	22.4 6.2 4.1 23.3 15.4 28.6
Infusions or transfusion reactions	Never happened Everyday Several times a week Once a month Several times a year Don’t know	288 235 58 407 333 330	17.4 14.2 3.5 24.7 20.2 20.0
Patients or their families’ complaints	Never happened Everyday Several times a week Once a month Several times a year Don’t know	128 545 434 223 167 154	7.8 33.0 26.3 13.5 10.1 9.3

### Response to patient safety incidents, teamwork, handoffs information exchange and communication openness

As detailed in Table 3, the mean score and standard deviation of participants’ responses to PSIs was 3.40 (*SD*: 0.742) whereas teamwork, handoffs and information exchange, and communication openness among healthcare professionals recorded scores of 4.18 (0.566), 3.88 (0.671) and 3.84 (0.667) respectively.

**Table 3:** Descriptive statistics of the participants’ Response to PSIs and other variables

Variable	Mean	SD
Response to PSIs Teamwork Handoffs and information exchange Communication openness	3.40 4.18 3.88 3.84	0.742 0.566 0.671 0.667

### Influence of teamwork, communication openness and handoffs and information exchange on health professional’s response to PSIs 

Table 4 shows the linear regression analyses of the predictive effects of teamwork, handoffs and information exchange and communications openness on the response to PSIs by health professionals. The model was significant, predicting 28.3% of the response to PSIs among healthcare professionals *(R*^*2*^ *= 0.283, F*_*(3, 1648)*_ *= 180.264, p < 0.05)*. When the various variables were examined for their contribution to the model, teamwork (*β* = 0.270, *p* < 0.05), handoffs and information exchange (*β* = 0.180, *p* < 0.05), and communication openness (*β* = 0.310, *p* < 0.05) were significant predictors of the model. An increase in teamwork (0.270 points) was noticed for a unit of increase in the mean score of response to PSIs by healthcare professionals. A unit increase in the mean score of handoffs and information exchange was associated with an increased response to PSIs by healthcare professionals by 0.180 points. Similarly, healthcare professionals who perceived an increased level of communication openness were 0.310 points likely to respond to PSIs.

**Table 4:** A linear regression model testing the relationship between Teamwork, Handoffs and information exchange, Communication openness and Response to PSIs

	B	SE	Beta	t	Sig.
(Constant)	− 0.181	0.158		-1.152	0.250
Teamwork	0.353	0.031	0.270	11.330	0.000
Handoffs and information exchange	0.198	0.026	0.180	7.603	0.000
Communication openness **R**^**2**^ **= 0.283, F**_**(3, 1648)**_ **= 180.264**, ***p*** **< 0.05**	0.348	0.027	0.310	13.005	0.000

a. *Dependent Variable: Response to PSIs*

## Discussion

This study aimed to identify the occurrence of PSIs, the level of teamwork, handoffs and information exchange, and communication openness among health professionals, and to examine predictors of response to PSIs. The daily frequencies of PSIs varied from 2.1% (pressure ulcers) to 33.0% (patient/family complaints) in healthcare facilities. This finding is similar to the results presented by Schwendimann et al. [53]. Despite variations in frequencies between in-hospital PSIs, they certainly have harmful impacts on patient outcomes and therefore the need for effective strategies to curtail them.

The study asserted that the main types of PSIs reported were associated with medication errors, surgery, and healthcare-related infections. Studies have shown that quality improvement interventions can lead to significant patient safety progress [54], and evidence of effective strategies is widely available [55]. For instance, hospitals can adopt individual or bundled interventions from other sectors, such as aviation, to reduce PSIs, and use patient safety practices as a key component [56, 57]. Vincent et al. [58] argue that to improve healthcare safety, comprehensive and balanced frameworks should be utilized to measure, monitor, and improve care safety. This includes fostering a safety culture about the most common types of PSIs. It is, therefore, important to put in place accurate monitoring of PSIs in healthcare facilities, and retrospective record reviews as evidence-based strategies to evaluate PSIs occurrence to reduce patient harm.

The finding of higher teamwork scores manifested in this current study is similar to other studies. This suggests that healthcare professionals work together consistently and are stable over time. With their significant role in healthcare delivery, teams with high levels of collaboration and communication have better patient safety scores and patient outcomes [59]. Further research may, however, be needed to determine if these scores reflect actual teamwork behaviours and if they impact patient outcomes. In healthcare, recent initiatives have been adopted globally to train providers on critical skills, such as communication, and team collaboration [60].

The findings of the study revealed handoffs and the exchange of information to be satisfactory in healthcare facilities. This is similar to the report in Jordan [61] and South Korea [62]. A human-centred approach that focuses on teamwork and communication can help to improve the efficiency and effectiveness of the handoff process [63]. These can include strategies such as regular training and practice in effective handoff communication. They can also include creating a culture of openness and encouraging healthcare team members to speak up when they have concerns. Implementing these tools and technologies can support effective handoff communication [64].

Though the study reported satisfactory teamwork and open communication which is supported by studies in Belgium [65], South Africa [66] and the USA [67], there was ironically a higher reported rate for some of the PSIs. This paradox of the “double-edged sword” of teamwork in healthcare possess a challenge to patient safety in PSIs reporting. This statement highlights the importance of transparency and learning cultures in healthcare organizations. This is where PSIs are seen as opportunities for growth and improvement, rather than evidence of failure. High-functioning teams are characterized by their ability to openly discuss and learn from errors, which leads to better patient outcomes. This concept is supported by research that shows the positive impact of systematic team training on patient safety, teamwork, and communication. By fostering a blame-free environment, healthcare organizations can create a culture of continuous learning and improvement.

Moreover, while open and positive communication styles are critical for building trust and cohesion among team members, they can also lead to complacency and a false sense of security. This results in increased PSI rates. It is also necessary, therefore, to highlight the importance of balancing open communication with rigorous systems, processes, and a culture of safety. This encourages reporting and learning from PSIs [68–70]. Additionally, healthcare teams must have an open and honest approach to PSI management and continuous learning. This is to ensure that PSIs are recognized, reported, and used as opportunities for improvement [71].

The study posited that PSIs occur in any healthcare setting, and open communication, efficient hand-over and teamwork enhance PSI response. This position is supported by Amaniyan et al. [15] and Baik et al. [34] who indicated that effective response to PSIs in healthcare requires a team effort and efficient communication. It has been noted that a high level of awareness and an “index of suspicion” when interpreting patient data is crucial in recognizing potential PSIs. Coordination and collaboration among team members can help manage PSIs and ensure timely and effective responses [72, 73].

### Limitations

The study used a cross-sectional approach, which means it only looks at data from one point in time and cannot establish causality. Again, the study relied on self-reported data from health professionals, which may not be completely accurate. There was, however, consistency in the distribution of data with existing literature. Additionally, the study used participant-reported measures of teamwork, handoffs, and communication openness, which may not be as reliable as other types of data. Finally, PSI rates may be low at the unit level to detect differences, even though they can have significant consequences for individual patients.

## Conclusion

The results of this study highlight the critical importance of effective communication, teamwork, and seamless handoffs in the hospital setting. There is no doubt that deficiencies in these areas are responsible for a significant portion of patient safety incidents. It is evident that enhancing collaborative teamwork among healthcare professionals and fostering a culture of openness are essential steps to reducing such incidents. Investing in training, protocols, and systems that facilitate smooth handoffs must be a priority for hospitals and healthcare organizations. They must also create an environment where healthcare workers feel empowered to communicate openly about potential risks and concerns. It is through addressing these root causes that we can create a safer healthcare environment for patients and support healthcare providers’ well-being as well. It is also important to recognize by creating a culture that encourages the response to errors and views them as opportunities for learning and improvement, rather than as failures, healthcare professionals can better identify and address potential problems, leading to better patient outcomes. It is also recommended that training on non-technical skills (such as ways to prevent adverse events) begin during regular education and in-service training as a requirement for the renewal of a licence to practice in healthcare facilities.

## Data Availability

The datasets used and/or analyzed during the current study are available from the corresponding author on reasonable request.

## Abbreviations

AHRQ: Agency for Healthcare Research and Quality
APES: Adverse Patient Event Scale
CHPS: Community-based Health Planning and Services
GHS: Ghana Health Service
HICs: High-income countries
HSOPS: Hospital Survey on Patient Safety Culture
ICU: Intensive care unit
IOM: Institute of Medicine
LMICs: Low-and Middle-Income Countries
PSIs: Patient Safety Incidents
SSA: Sub-Saharan Africa

## References

[1] World Health Organization. World alliance for patient safety: forward programme 2005. 2004.

[2] Hessels A, Paliwal M, Weaver SH, Siddiqui D, Wurmser TA (2019). Impact of patient safety culture on missed nursing care and adverse patient events. J Nurs Care Qual.

[3] Panagioti M, Khan K, Keers RN, Abuzour A, Phipps D, Kontopantelis E et al. Prevalence, severity, and nature of preventable patient harm across medical care settings: systematic review and meta-analysis. BMJ. 2019;366. 10.1136/bmj.l4185 PMC6939648 31315828

[4] Sunshine JE, Meo N, Kassebaum NJ, Collison ML, Mokdad AH, Naghavi M (2019). Association of adverse effects of medical treatment with mortality in the United States: a secondary analysis of the global burden of diseases, injuries, and risk factors study. JAMA Netw Open.

[5] Hadi U, Pudjiraharjo WJ, Damayanti NA, Chalidyanto D, Rochmah T. How does team performance management influence the knowledge in implementing patient safety programs in the hospital work unit, Indonesia? Syst Rev Pharm. 2020;11(11).

[6] Mikos M, Budzowska J, Banaś T, Kiedik D, Sygit K, Cipora E (2022). Civil lawsuits as an Indicator of adverse outcomes in Healthcare. Int J Environ Res Public Health.

[7] Elliott RA, Camacho E, Jankovic D, Sculpher MJ, Faria R (2021). Economic analysis of the prevalence and clinical and economic burden of medication error in England. BMJ Qual Saf.

[8] Nauman J, Soteriades ES, Hashim MJ, Govender R, Al Darmaki RS, Al Falasi RJ et al. Global incidence and mortality trends due to adverse effects of medical treatment, 1990–2017: a systematic analysis from the global burden of diseases, injuries and risk factors study. Cureus. 2020;12(3). 10.7759/cureus.7265 PMC7075477 32195071

[9] Domer G, Gallagher TM, Shahabzada S, Sotherland J, Paul EN, Kumar KN et al. Patient safety: preventing patient harm and building capacity for patient safety. Contemporary topics in Patient Safety-Volume 1. IntechOpen; 2021.

[10] Maher A, Ayoubian A, Rafiei S, Sheibani Tehrani D, Mostofian F, Mazyar P (2019). Developing strategies for patient safety implementation: a national study in Iran. Int J Health Care Qual Assur.

[11] Ocloo J, Garfield S, Franklin BD, Dawson S (2021). Exploring the theory, barriers and enablers for patient and public involvement across health, social care and patient safety: a systematic review of reviews. Health Res Policy Syst.

[12] Konlan KD, Shin J (2022). The status and the factors that influence patient safety in health care institutions in Africa: a systematic review. PLOS Glob Public Health.

[13] Lee E, Kim Y (2020). The relationship of moral sensitivity and patient safety attitudes with nursing students’ perceptions of disclosure of patient safety incidents: a cross-sectional study. PLoS ONE.

[14] Burke JR, Downey C, Almoudaris AM (2022). Failure to rescue deteriorating patients: a systematic review of root causes and improvement strategies. J Patient Saf.

[15] Amaniyan S, Faldaas BO, Logan PA, Vaismoradi M (2020). Learning from patient safety incidents in the emergency department: a systematic review. J Emerg Med.

[16] Simsekler ME, Gurses AP, Smith BE, Ozonoff A (2019). Integration of multiple methods in identifying patient safety risks. Saf Sci.

[17] Smith AF, Plunkett E (2019). People, systems and safety: resilience and excellence in healthcare practice. Anaesthesia.

[18] Yan L, Tan J, Chen H, Yao L, Li Y, Zhao Q (2022). Experience and support of chinese healthcare professionals as second victims of patient safety incidents: a cross-sectional study. Perspect Psychiatr Care.

[19] Kim Y, Lee H (2020). Nurses’ experiences with disclosure of patient safety incidents: a qualitative study. Risk Manag Healthc Policy.

[20] Kumbi M, Hussen A, Lette A, Nuriye S, Morka G (2020). Patient Safety Culture and Associated factors among Health Care Providers in Bale Zone Hospitals, Southeast Ethiopia: an institutional based cross-sectional study. Drug Healthc Patient Saf.

[21] Kruk ME, Gage AD, Arsenault C, Jordan K, Leslie HH, Roder-DeWan S (2018). High-quality health systems in the Sustainable Development Goals era: time for a revolution. Lancet Glob Health.

[22] Shirey MR, White-Williams C, Hites L (2019). Integration of authentic leadership lens for building high performing interprofessional collaborative practice teams. Nurs Adm Q.

[23] Michalsen A, Long AC, Ganz FD, White DB, Jensen HI, Metaxa V (2019). Interprofessional shared decision-making in the ICU: a systematic review and recommendations from an expert panel. Crit Care Med.

[24] Khoshakhlagh AH, Khatooni E, Akbarzadeh I, Yazdanirad S, Sheidaei A (2019). Analysis of affecting factors on patient safety culture in public and private hospitals in Iran. BMC Health Serv Res.

[25] Alenezi A, Pandaan RPM, Almazan JU, Pandaan IN, Casison FS, Cruz JP (2019). Clinical practitioners’ perception of the dimensions of patient safety culture in a government hospital: a one-sample correlational survey. J Clin Nurs.

[26] Kakemam E, Albelbeisi AH, Davoodabadi S, Ghafari M, Dehghandar Z, Raeissi P (2022). Patient safety culture in iranian teaching hospitals: baseline assessment, opportunities for improvement and benchmarking. BMC Health Serv Res.

[27] Azyabi A, Karwowski W, Davahli MR (2021). Assessing patient safety culture in hospital settings. Int J Environ Res Public Health.

[28] Kakemam E, Gharaee H, Rajabi MR, Nadernejad M, Khakdel Z, Raeissi P (2021). Nurses’ perception of patient safety culture and its relationship with adverse events: a national questionnaire survey in Iran. BMC Nurs.

[29] Modaresnezhad M, Andrews MC, Mesmer-Magnus J, Viswesvaran C, Deshpande S (2021). Anxiety, job satisfaction, supervisor support and turnover intentions of mid-career nurses: a structural equation model analysis. J Nurs Manag.

[30] Kakemam E, Hajizadeh A, Azarmi M, Zahedi H, Gholizadeh M, Roh YS (2021). Nurses’ perception of teamwork and its relationship with the occurrence and reporting of adverse events: a questionnaire survey in teaching hospitals. J Nurs Manag.

[31] Han JH, Roh YS (2020). Teamwork, psychological safety, and patient safety competency among emergency nurses. Int Emerg Nurs.

[32] Baker DP, Day R, Salas E (2006). Teamwork as an essential component of high-reliability organizations. Health Serv Res.

[33] Etherington C, Burns JK, Kitto S, Brehaut JC, Britton M, Singh S (2021). Barriers and enablers to effective interprofessional teamwork in the operating room: a qualitative study using the theoretical domains Framework. PLoS ONE.

[34] Baik D, Zierler B (2019). Clinical nurses’ experiences and perceptions after the implementation of an interprofessional team intervention: a qualitative study. J Clin Nurs.

[35] Tocco Tussardi I, Benoni R, Moretti F, Tardivo S, Poli A, Wu AW (2021). Patient safety in the eyes of aspiring healthcare professionals: a systematic review of their attitudes. Int J Environ Res Public Health.

[36] Hüner B, Derksen C, Schmiedhofer M, Lippke S, Riedmüller S, Janni W (2023). Reducing preventable adverse events in obstetrics by improving interprofessional communication skills–results of an intervention study. BMC Pregnancy Childbirth.

[37] Chen Y, Gong Y (2022). Teamwork and patient safety in Intensive Care Units: Challenges and Opportunities. Stud Health Technol Inform.

[38] Lee SH, Phan PH, Dorman T, Weaver SJ, Pronovost PJ (2016). Handoffs, safety culture, and practices: evidence from the hospital survey on patient safety culture. BMC Health Serv Res.

[39] Suclupe S, Kitchin J, Sivalingam R, McCulloch P (2023). Evaluating patient identification Practices during Intrahospital Transfers: a human factors Approach. J Patient Saf.

[40] Varughese AM, Buck DW, Lane-Fall BM, Heitmiller ES. Quality improvement in anaesthesia practice and patient safety. Miller’s Anesth 9th Ed Phila Elsevier Inc. 2020;84–104.

[41] Appelbaum R, Martin S, Tinkoff G, Pascual JL, Gandhi RR (2021). Eastern association for the surgery of trauma – quality, patient safety, and outcomes committee - transitions of care: healthcare handoffs in trauma. Am J Surg.

[42] O’Donovan R, McAuliffe E (2020). A systematic review exploring the content and outcomes of interventions to improve psychological safety, speaking up and voice behaviour. BMC Health Serv Res.

[43] Aldawood F, Kazzaz Y, AlShehri A, Alali H, Al-Surimi K (2020). Enhancing teamwork communication and patient safety responsiveness in a paediatric intensive care unit using the daily safety huddle tool. BMJ Open Qual.

[44] Spector PE (2019). Do not Cross Me: optimizing the Use of cross-sectional designs. J Bus Psychol.

[45] World Health Organization. The state of the health workforce in the WHO African Region, 2021 ISBN. 2021.

[46] Asamani JA, Ismaila H, Plange A, Ekey VF, Ahmed AM, Chebere M (2021). The cost of health workforce gaps and inequitable distribution in the Ghana health service: an analysis towards evidence-based health workforce planning and management. Hum Resour Health.

[47] Cochran WG. Sampling techniques. John Wiley & Sons; 1977.

[48] Laschinger HKS, Leiter MP (2006). The impact of nursing work environments on patient safety outcomes: the mediating role of burnout engagement. JONA J Nurs Adm.

[49] Aiken LH, Sloane D, Griffiths P, Rafferty AM, Bruyneel L, McHugh M (2017). Nursing skill mix in european hospitals: a cross-sectional study of the association with mortality, patient ratings, and quality of care. BMJ Qual Saf.

[50] Sorra J, Yount N, Famolaro T, Gray L. AHRQ Hospital survey on patient safety culture version 2.0: user’s guide. Rockv Agency Healthc Res Qual. 2019.

[51] Lee SE, Dahinten VS (2021). Adaptation and validation of a korean-language version of the revised hospital survey on patient safety culture (K-HSOPSC 2.0). BMC Nurs.

[52] Suryani L, Letchmi S, Said FBM (2022). Cross-culture adaptation and validation of the indonesian version of the Hospital Survey on Patient Safety Culture (HSOPSC 2.0). Belitung Nurs J.

[53] Schwendimann R, Blatter C, Dhaini S, Simon M, Ausserhofer D (2018). The occurrence, types, consequences and preventability of in-hospital adverse events–a scoping review. BMC Health Serv Res.

[54] Portela MC, Pronovost PJ, Woodcock T, Carter P, Dixon-Woods M (2015). Republished: how to study improvement interventions: a brief overview of possible study types. Postgrad Med J.

[55] Shekelle PG, Pronovost PJ, Wachter RM, McDonald KM, Schoelles K, Dy SM (2013). The top patient safety strategies that can be encouraged for adoption now. Ann Intern Med.

[56] Luangasanatip N, Hongsuwan M, Limmathurotsakul D, Lubell Y, Lee AS, Harbarth S et al. Comparative efficacy of interventions to promote hand hygiene in hospital: systematic review and network meta-analysis. BMJ. 2015;351. 10.1136/bmj.h3728 PMC4517539 26220070

[57] Mueller SK, Sponsler KC, Kripalani S, Schnipper JL (2012). Hospital-based medication reconciliation practices: a systematic review. Arch Intern Med.

[58] Vincent C, Neale G, Woloshynowych M (2001). Adverse events in british hospitals: preliminary retrospective record review. BMJ.

[59] Ekici D, Yildirim A, Durmuş Sç (2018). The level of collaboration amongst nurses in Turkey. Int Nurs Rev.

[60] Prineas S, Mosier K, Mirko C, Guicciardi S. Non-technical skills in healthcare. Textb Patient Saf Clin Risk Manag. 2021;413–34. 36315753

[61] Dalky HF, Al-Jaradeen RS, AbuAlRrub RF (2020). Evaluation of the Situation, background, Assessment, and recommendation handover Tool in improving communication and satisfaction among jordanian nurses working in Intensive Care Units. Dimens Crit Care Nurs.

[62] Kim JH, Lee JL, Kim EM (2021). Patient safety culture and handoff evaluation of nurses in small and medium-sized hospitals. Int J Nurs Sci.

[63] Muinga N, Paton C, Gicheha E, Omoke S, Abejirinde IOO, Benova L (2021). Using a human-centred design approach to develop a comprehensive newborn monitoring chart for inpatient care in Kenya. BMC Health Serv Res.

[64] Ng GWY, Pun JKH, So EHK, Chiu WWH, Leung ASH, Stone YH (2017). Speak-up culture in an intensive care unit in Hong Kong: a cross-sectional survey exploring the communication openness perceptions of chinese doctors and nurses. BMJ Open.

[65] von Knorring M, Griffiths P, Ball J, Runesdotter S, Lindqvist R (2020). Patient experience of communication consistency amongst staff is related to nurse–physician teamwork in hospitals. Nurs Open.

[66] Nyalunga SLN, Ndimande JV, Ogunbanjo GA, Masango-Makgobela A, Bogongo T (2019). Perceptions of community health workers on their training, teamwork and practice: a cross-sectional study in Tshwane district, Gauteng, South Africa. South Afr Fam Pract.

[67] Herzberg S, Hansen M, Schoonover A, Skarica B, McNulty J, Harrod T (2019). Association between measured teamwork and medical errors: an observational study of prehospital care in the USA. BMJ Open.

[68] O’Donovan R, Ward M, De Brún A, McAuliffe E (2019). Safety culture in health care teams: a narrative review of the literature. J Nurs Manag.

[69] Provan DJ, Woods DD, Dekker SW, Rae AJ (2020). Safety II professionals: how resilience engineering can transform safety practice. Reliab Eng Syst Saf.

[70] Kolbe M, Eppich W, Rudolph J, Meguerdichian M, Catena H, Cripps A (2020). Managing psychological safety in debriefings: a dynamic balancing act. BMJ Simul Technol Enhanc Learn.

[71] Ridley CH, Al-Hammadi N, Maniar HS, Abdallah AB, Steinberg A, Bollini ML (2021). Building a collaborative culture: focus on psychological safety and error reporting. Ann Thorac Surg.

[72] Kennedy-Metz LR, Barbeito A, Dias RD, Zenati MA (2022). Importance of high-performing teams in the cardiovascular intensive care unit. J Thorac Cardiovasc Surg.

[73] Müller M, Jürgens J, Redaèlli M, Klingberg K, Hautz WE, Stock S (2018). Impact of the communication and patient hand-off tool SBAR on patient safety: a systematic review. BMJ Open.

